# The Association between Internalizing Symptoms and Witnessing School Bullying and Defending Behavior: An Analysis of Gender Differences among Elementary and Middle School Students

**DOI:** 10.3390/children10071199

**Published:** 2023-07-11

**Authors:** Diana M. Doumas, Aida Midgett, Matt Peck

**Affiliations:** 1 Institute for the Study of Behavioral Health and Addiction, Boise State University, 1910 University Drive, Boise, ID 83725, USA; aidamidgett@boisestate.edu; 2 Department of Counselor Education, Boise State University, 1910 University Drive, Boise, ID 83725, USA; mattpeck@boisestate.edu

**Keywords:** bullying, bystander, defending behavior, depressive symptoms, social anxiety, elementary school, middle school

## Abstract

Bullying is a significant public health concern that begins as early as elementary school and peaks in middle school. Although researchers have demonstrated the relationship between internalizing symptoms and being a target of bullying, there is limited research examining the association between internalizing symptoms and witnessing school bullying and defending targets or gender differences in these relationships. In this cross-sectional study, we examined gender as a moderator of the relationships between internalizing symptoms (e.g., depressive symptoms and social anxiety) and witnessing school bullying and defending behavior in a sample of elementary and middle school students (*N* = 126; 51.6% female; 3rd–8th grade). Results demonstrated that witnessing school bullying was a significant predictor of depressive symptoms. For social anxiety, the gender x witnessing school bullying interaction was significant for social avoidance and distress (SAD), such that among female students, SAD was positively related to witnessing school bullying. In contrast, the gender x defending behavior interaction was significant for fear of negative evaluation (FNE), such that among male students, FNE was positively related to defending behavior. Findings suggest bullying prevention should incorporate bystander training programs that include a focus on gender differences in social anxiety associated with being a bullying bystander.

## 1. Introduction

Bullying has been defined as “the repetitive, intentional hurting of one person or group by another person or group, where the relationship involves an imbalance of power” [1]. According to United States (U.S.) national survey data, among students aged 12–18, 22.2% report bullying victimization [2]. School bullying victimization is reported by elementary school students (22%) [3] and peaks in middle school (27.6%) [2], suggesting elementary and middle school youth are the most vulnerable to bullying victimization. Additionally, 25.5% of females report school bullying victimization relative to 19.1% of males [2]. Further, results from a meta-analysis investigating the impact of bullying victimization on youth demonstrate that targets report a wide range of socio-emotional consequences, including anxiety, post-traumatic stress, depressive symptoms, suicidal ideation and attempts, and poor mental and physical health [4].

### 1.1. Bullying Bystanders 

A bystander can be defined as someone who observes bullying but is not involved in bullying perpetration and is not the target of bullying [5]. Students who witness bullying can act in several ways, including directly helping the bully by joining in the bullying behavior as “assistants”, promoting the bullying and motivating the bully as “reinforcers”, ignoring or leaving the bullying situations as “outsiders”, or doing something to interrupt or stop the bullying as “defenders” [6]. Research suggests that up to 80% of youth observe bullying behavior at school [7]. The Bystander Intervention Model [8] provides a conceptual framework for understanding the defending behavior among bystanders. The Bystander Intervention Model suggests that bystanders must move through a series of five sequential steps to defend targets: (a) notice the bullying event, (b) interpret the bullying event as an emergency that requires assistance, (c) accept responsibility for intervening in the observed bullying situation, (d) know how to intervene in the bullying situation, and (e) intervene in the bullying situation. Research with middle school students demonstrates that each step of the Bystander Intervention Model is positively associated with defending behavior [9]. A recent review of the literature examining the factors that contribute to students taking action as “defenders” indicates that altruism, social competence, self-esteem, self-efficacy, perspective taking, and empathy are all positively related to defending behavior [10]. There is, however, a need to extend the literature beyond factors associated with bystander intervention to research investigating the negative mental health outcomes related to witnessing bullying and defending behavior. 

### 1.2. Mental Health Outcomes for Bystanders

Researchers have extended the examination of mental health consequences among targets of school bullying to mental health risks experienced by student bystanders. For example, internalizing symptoms are positively associated with witnessing school bullying [11,12]. One explanation for this association is that bystanders may feel helpless [11], anxious about their own safety, or experience vicarious trauma [13] when observing bullying. Similar to witnessing bullying, depressive symptoms [7,11,14,15], anxiety [11,14], and social anxiety [7,15] are also related to intervening in bullying situations. Research indicates that the decision to intervene in bullying situations is impacted by social norms [16]. Internalizing symptoms associated with defending behavior may be related to pro-bullying norms, with “defenders” becoming socially isolated as a result of intervening when they witness bullying [7]. Further, bystanders may fear retaliation when defending targets [17].

### 1.3. Gender Differences in Witnessing Bullying and Defending Behavior

Research indicates that rates of witnessing bullying [14] and defending behavior [18] are higher for female students relative to male students. Research examining the Bystander Intervention Model provides evidence that female students are more likely to notice bullying and to understand that bullying is a situation that needs to be acted upon [19]. Researchers have also identified gender differences when investigating mental health risks among bullying bystanders. Specifically, female students report depression and social anxiety related to witnessing bullying, whereas males do not [15]. This gender difference may be related to developmentally higher levels of empathy and perspective taking among females in this age group [20]. Additionally, for females, internalizing symptoms associated with witnessing bullying may be associated with interpreting bullying as a serious situation that needs intervention [19]. In contrast, males report depression [14,15] and anxiety [14] related to defending, while depression and anxiety are not related to defending among females [14,15]. It is possible that defending behavior is positively associated with internalizing symptoms among males, as males may use aggressive behavior when defending [14] and peers are more likely to socially reject males who defend targets [6].

Although researchers have identified gender differences in internalizing symptoms among bystanders [14,15], each study has its limitations. Lambe et al. (2017) [14] assessed depression and anxiety by combining two items to create an internalizing scale. The internal consistency of the scale was low (a = 0.64), potentially due to combining two distinct constructs. Additionally, Lambe et al.’s data analysis did not include bullying victimization as a control variable. Further, Lambe et al. investigated the impact of general anxiety, but not social anxiety, on defending behavior. In contrast, although Midgett et al. (2021) [15] controlled for experiences of bullying victimization and investigated social anxiety specifically, the constructs of social avoidance and distress (SAD) and fear of negative evaluation (FNE) were combined. Further, the study sample was limited to sixth grade students.

### 1.4. The Current Study

The limited research on gender differences in internalizing symptoms among bystanders suggests that emotional outcomes for female and male students may be different. The aim of this study was to extend the research on gender differences by investigating these relationships among elementary and middle school students with the goal of providing information to guide prevention programming. We utilized cross-sectional methodology to investigate the association between internalizing symptoms and witnessing school bullying and defending behavior. To extend the literature specific to social anxiety, we examined two constructs: social avoidance and distress (SAD) and fear of negative evaluation (FNE). Our hypotheses were (a) gender would moderate the relationship between depressive symptoms and witnessing bullying and defending behavior and (b) gender would moderate the relationship between social anxiety (i.e., SAD and FNE) and witnessing bullying and defending behavior.

## 2. Materials and Methods

### 2.1. Participants and Procedures

Participants were 126 students (51.6% female; 48.4% male) enrolled in two elementary schools and one middle school in the northwest region of the U.S. The age range of participants was ages 7–14 (M = 10.45 and SD = 1.71). In total, 88 students (69.8%) were in elementary school (grades 3–5) and 38 students (30.2%) were in middle school (grades 6–8). The sample was predominantly White (63.7%), with 12.9% identifying as more than one race, 11.3% as Hispanic, 3.2% as Black, 1.6% as Asian American, and 7.3% as Other.

The research team recruited all students in the third through fifth grade from two elementary schools and sixth through eighth grade from one middle school (*N* = 468) to participate in this study. The school sent an email to parents/guardians that included study information and an informed consent form. Additionally, during classroom time, the school counselor provided consent forms that students could take to their parents/guardians for a signature. Students with parent/guardian signed consent forms provided assent prior to beginning data collection procedures. Parental/guardian consent was obtained for 272 students (58.1%). A total of 253 (54.1%) students assented to participate. Study procedures were implemented during class time. The questionnaire took 20 min to administer. Incentives included pizza at the completion of study procedures. For this study, we included students who reported witnessing bullying in the month prior to this study (*N* = 126; 49.8%).

### 2.2. Measures

Demographic Survey. This survey included questions about gender, grade, age, and race/ethnicity. Participants indicated their gender, grade, and age through open-ended questions and provided their race/ethnicity through response choices.

Witnessing Bullying. The global Olweus Bullying Questionnaire [21] was used to measure the frequency of witnessing bullying in the past 30 days. The global bystander item was used. The item was rated on a 5-point Likert Scale with anchors of 0 (I Have Not) to 4 (Several Times a Week). The questionnaire has a good construct validity [22].

Defending Behavior. The 3-item Defender Subscale of the Participants Roles Questionnaire (PRQ) [23] was used to measure defending behaviors. The items were rated on a 3-point Likert Scale with anchors of 0 (Never) to 2 (Often). Researchers have demonstrated a good construct validity [6] and moderate to good internal reliability (α = 0.79–0.93) [23,24]. For the current sample, α = 0.80.

Depressive Symptoms. The 20-item Center for Epidemiological Studies Depression Scale for Children (CES-DC) [25] was used to measure depressive symptoms. The items were rated on a 4-point Likert Scale with anchors of 0 (Not at All) to 3 (A Lot). Scale psychometrics include a demonstrated construct validity [25], good test–retest reliability [26], and good internal reliability (α = 0.89) [27]. For the current sample, α = 0.90.

Social Anxiety. Social anxiety was measured using the 22-item Social Anxiety Scale for Adolescents (SAS-A) [28]. We used the 10-item Social Avoidance and Distress Scale (SAD) and the 8-item Fear of Negative Evaluation Scale (FNE). The SAD Scale measures social avoidance of peers and social distress in new and typical situations; the FNE Scale measures anxiety related to peer’s negative evaluations. The items were rated on a 5-point Likert Scale with anchors of 0 (Not at All) to 4 (All the Time). The scale has a good construct validity [28,29], moderate test–retest reliability [30], and good internal reliability (α = 0.76–0.91) [29]. For the current sample, for SAD, α = 0.89, and for FNE, α = 0.80.

### 2.3. Statistical Analyses

IBM SPSS Statistics for Windows, Version 28.0 was used to conduct all analyses. Data were examined for missing values and we used linear interpolation to impute missing data [31]. We examined all variables for normality, with skew and kurtosis values of −2 and +2 considered as acceptable [32]. We also calculated bivariate correlations to examine multicollinearity among predictor variables and associations among predictor and dependent variables. We considered variance inflation factor (VIF) values below 10 as acceptable [33]. We then conducted three hierarchical regression analyses, with moderation tested through interaction effects. Because our equations contained interaction terms, we mean centered predictor variables to decrease issues related to multicollinearity [34]. In Step 1, we entered the control variables bullying victimization and grade. In Step 2, we entered gender, witnessing school bullying, and defending behavior. In Step 3, we entered the interaction terms gender x witnessing school bullying and gender x defending behavior. For significant interactions, we examined the direction and magnitude by plotting simple slopes [34]. All analyses were considered significant at *p* < 0.05. We set effect size (*R*^2^) magnitude values at small = 0.01, medium = 0.09, and large = 0.25 [35].

### 2.4. Power Calculations

We used G*Power 3.1.3 [36] to conduct a power analysis to determine the sample size. For a regression model with five tested predictors and seven total predictors, a sample size of 92 is required for a power of ≥0.80 to detect a medium effect size for *R*^2^ increases with a 0.05 alpha level. Thus, our sample of 126 participants provided adequate power for our analyses.

## 3. Results

### 3.1. Preliminary Analyses

Means and standard deviations are presented in Table 1. For all variables, skew and kurtosis were acceptable; skew ranged from −0.38 to 1.06 and kurtosis ranged from −0.10 to −1.05. Bivariate correlations are presented in Table 2. Multicollinearity was also acceptable; VIF ranged between 1.01 and 1.82.

### 3.2. Depressive Symptoms

Table 3 presents the regression analysis for depressive symptoms. The adjusted *R*^2^ for the model was *R*^2^ = 0.13. The effect size is medium. Witnessing school bullying was a significant predictor of depressive symptoms (*p* < 0.03), with findings demonstrating a positive association. In contrast, defending behavior was not a significant predictor of depressive symptoms (*p* = 0.63). Additionally, neither the gender x witnessing school bullying (*p* = 0.27) nor the gender x defending behavior (*p* = 0.51) interaction terms were significant, suggesting gender was not a significant moderator.

### 3.3. Social Avoidance and Distress (SAD)

**Figure 1:**
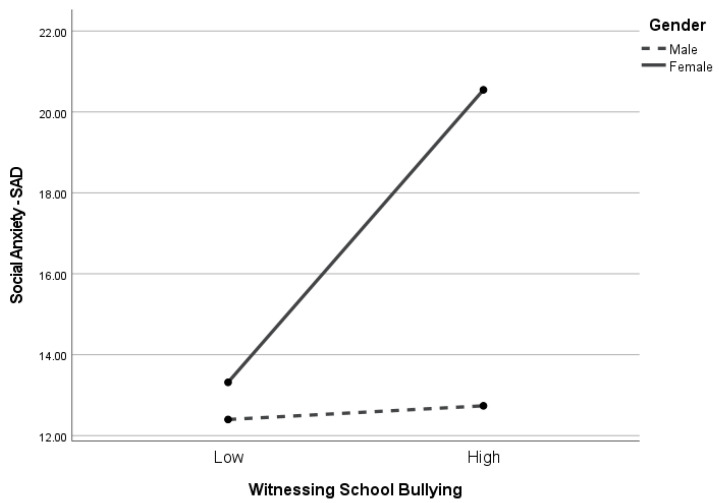
Simple slopes for social distress and avoidance and witnessing school bullying by gender. Note. The figure illustrates the direction and degree of the significant interaction effect (i.e., gender x SAD) depicted by simple slopes for witnessing school bullying (*p* = 0.005). SAD was significantly associated with witnessing school bullying among female students (*p* = 0.006) but not among male students (*p* = 0.26).

Table 4 presents the regression analysis for SAD. The adjusted *R*^2^ for the model was *R*^2^ = 0.09. The effect size is medium. Neither witnessing school bullying (*p* = 0.23) nor defending behavior (*p* = 0.77) were significant predictors of SAD. Additionally, the gender x defending behavior interaction was not significant (*p* = 0.31). However, the gender x witnessing school bullying interaction term was significant (*p* < 0.005). As seen in Figure 1, among female students, witnessing school bullying was positively associated with SAD (*p* < 0.006), whereas the association between witnessing school bullying and SAD was not significant among male students (*p* = 0.26).

### 3.4. Fear of Negative Evaluation (FNE)

**Figure 2:**
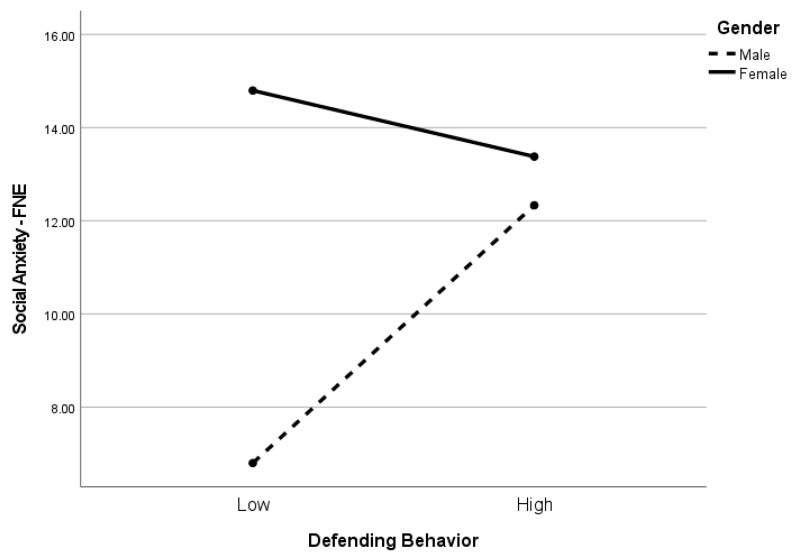
Simple slopes for fear of negative evaluation and defending behavior by gender. Note. The figure illustrates the direction and degree of the significant interaction effect (i.e., gender x FNE) depicted by simple slopes for defending behavior (*p* = 0.05). FNE was significantly associated with defending behavior among male students (*p* = 0.04) but not among female students (*p* = 0.49).

Table 5 presents the regression analysis for FNE. The adjusted *R*^2^ for the model was *R*^2^ = 0.13. The effect size is medium. Neither witnessing school bullying (*p* = 0.22) nor defending behavior (*p* = 0.34) were significant predictors of FNE. Additionally, the gender x witnessing school bullying interaction (*p* = 0.12) was not significant. However, the gender x defending behavior interaction term was significant (*p* < 0.05). As seen in Figure 2, among male students, defending behavior was positively associated with FNE (*p* < 0.04), whereas the association between defending behavior and FNE was not significant among female students (*p* = 0.49).

## 4. Discussion

The purpose of this study was to examine gender differences in internalizing symptoms among elementary and middle school students who witness bullying and act as “defenders”. Results indicate that depressive symptoms were positively associated with witnessing school bullying for both male and female students. In contrast, for social anxiety, we found a significant interaction effect for gender, revealing gender differences in the association between social anxiety and both witnessing school bullying and defending behavior. Gender effects, however, were different depending on the type of social anxiety reported. Specifically, for females, SAD was positively related to witnessing school bullying; for males, defending behavior was positively related to FNE.

Results are not consistent with our first hypothesis that we would find significant gender differences in the relationship between depressive symptoms and witnessing school bullying and defending behavior. Prior research indicates that for females, depression is associated with witnessing school bullying, whereas for males, depression is associated with defending behavior [14,15]. Although our results are consistent with prior research for females, we did not find evidence that depression was associated with defending behavior for males. Instead, similarly to females, depression was positively associated with witnessing school bullying. Thus, both female and male students in the current study experienced depressive symptoms related to witnessing school bullying, but not related to defending behavior. The discrepancy in our results may be due to differences in sample characteristics compared to prior studies. Specifically, the prior samples included sixth grade students only [15] or fourth through twelfth grade students [14]. Additionally, inclusion criteria varied, with one study including all students regardless of bystander status [15] and another study including students who witnessed bullying but were not involved in bullying perpetration and had not experienced bullying victimization [14].

Consistent with our second hypothesis, we did find evidence to support gender as a moderator of the relationship between social anxiety and witnessing school bullying and defending behavior. Results parallel previous research examining gender differences in anxiety and witnessing bullying [15] and defending behavior [14,15] while extending the literature by including SAD and FNE as two distinct constructs of social anxiety. Findings from the current study demonstrate that among female students, SAD was positively related to witnessing school bullying, whereas among male students, FNE was positively related to defending behavior. One explanation for these gender differences is that female bystanders may experience social distress when they observe a bullying situation due to higher levels of empathy and perspective taking [20]. Further, females are more likely than males to understand that bullying is associated with negative outcomes [19]. Thus, when witnessing school bullying, females may experience psychological co-victimization [37], leading to higher levels of SAD in females relative to males. Additionally, research indicates bullying victimization may increase the anticipatory anxiety of being bullied again, which, in turn, increases the risk of developing social anxiety [38]. A similar pattern may be true for witnessing bullying, in which female bystanders may experience higher rates of anticipatory anxiety related to witnessing bullying due to heightened perspective taking, empathy, and understanding of the consequences of bullying. In contrast, males may experience higher levels of social evaluative anxiety when defending targets as males are more likely to use maladaptive strategies (e.g., aggressive behavior) relative to female students who generally use pro-social strategies (e.g., comforting targets or reporting bullying to adults at school) [39]. Further, peers are more likely to socially reject males who take action to defend students in bullying situations [6]. Thus, males may experience fear of negative evaluation due to using maladaptive forms of defending behavior and the associated social rejection from their peer group [15].

As for limitations, our research design was cross-sectional; future research using a longitudinal design is necessary to examine causality. Further, although we recruited students from three schools, all schools were recruited from the same region in the U.S. Additionally, the sample size was small. To increase generalizability, research with larger samples, including participants from a wide range of geographical locations, is needed. Finally, although the inclusion of both elementary and middle school students is a strength of this study, there are developmental differences between elementary and middle school students. Although we did control for grade in the analyses, and grade was not a significant predictor of any of the outcome variables, there are other cognitive, emotional, and social characteristics that were not addressed in this study.

Findings from the current study have several implications for practice. First, 49.8% of students in the current sample reported witnessing school bullying in the past month. Further, results indicate that both witnessing school bullying and defending behavior are positively related to depression and social anxiety, over and above being a target. Thus, the impact of bullying extends to bullying bystanders, with one half of students at risk for experiencing depression and social anxiety related to witnessing and/or intervening in school bullying. These data highlight the need for mental health and school professionals to assess and address internalizing symptoms among bystanders as part of bullying prevention at the elementary and middle school levels.

Further, findings from the current study reveal important gender differences for bystanders. Specifically, for female students, internalizing symptoms was positively related to witnessing school bullying only. In contrast, for male students, internalizing symptoms was positively related to both witnessing school bullying and defending behavior. Because 80% of students observe bullying [7], mental health and school professionals need to focus on bystanders’ mental health needs when implementing bullying prevention programs. Results from this study indicate that it is important for mental health and school professionals to recognize that students who report witnessing school bullying should be screened for internalizing symptoms. Additionally, mental health and school professionals need to understand that male and female student bystanders have different experiences. Female students may benefit from identifying feelings of both depression and social avoidance and distress related to witnessing school bullying. For males, in addition to providing coping skills, males may also benefit from learning skills that they can utilize when they observe bullying. Training male students to use pro-social skills may minimize the fear of negative evaluation associated with defending behavior. Additionally, creating a culture that supports defending behavior may decrease evaluation anxiety. Research indicates that when defending targets is perceived as the school norm, students are more likely to intervene in bullying situations [40], which may be particularly important for males who believe they will be negatively evaluated if they defend targets.

Bullying prevention programs, including comprehensive, school-wide programs with bystander training components [41] and stand-alone bullying bystander interventions [42,43], are effective in decreasing bullying behavior among elementary and middle school students. Additionally, research indicates that both comprehensive bullying programs with a peer focus [44] and stand-alone bystander programs [42,45] are effective in the decrease of internalizing symptoms among youth in this age group. Therefore, implementing school-based programs that focus on bystander training may be effective implementation strategies for decreasing internalizing symptoms. Tailoring programs to address differing needs of female and male students is also an important implementation consideration.

## 5. Conclusions

Findings from the current study indicate that for both female and male students, depressive symptoms are positively associated with witnessing school bullying. Gender differences related to social anxiety suggest that for females, witnessing school bullying is positively related to social avoidance and distress. In contrast, defending behavior is positively related to fear of negative evaluation for males. Results underscore the need for mental health and school professionals to attend to gender differences when implementing bullying bystander interventions to decrease internalizing symptoms among bystanders.

## Figures and Tables

**Table 1:** Means and Standard Deviations by Gender.

Variable	Gender		Total Sample
	Female	Male	
Depressive Symptoms	28.72 (13.75)	23.65 (12.96)	26.26 (13.56)
Social Anxiety—SAD	15.65 (10.34)	12.52 (10.75)	14.14 (10.64)
Social Anxiety—FNE	14.08 (9.72)	10.07 (10.35)	12.14 (10.12)
Witnessing Bullying	1.95 (1.18)	2.03 (1.24)	1.99 (1.20)
Defending Behavior	3.72 (1.81)	4.05 (1.79)	3.88 (1.80)

**Table 2:** Bivariate Correlations by Gender.

**Variable**	**Females**				
	**1**	**2**	**3**	**4**	**5**
1. Depressive Symptoms	-				
2. Social Anxiety—SAD	0.61 **	-			
3. Social Anxiety—FNE	0.67 **	0.68 **	-		
4. Witnessing Bullying	0.36 **	0.42 **	0.26 *	-	
5. Defending Behavior	0.10	0.00	−0.10	0.08	-
**Variable**	**Males**				
	**1**	**2**	**3**	**4**	**5**
1. Depressive Symptoms	-				
2. Social Anxiety—SAD	0.66 **	-			
3. Social Anxiety—FNE	0.66 **	0.77 **	-		
4. Witnessing Bullying	0.26 *	0.01	0.15	-	
5. Defending Behavior	0.04	0.14	0.27 *	0.25	-

* *p* < 0.05, ** *p* < 0.01.

**Table 3:** Hierarchical Regression Analyses for Depressive Symptoms.

Predictor	Δ*R*^2^	B	SE B	β	95% CI
Step 1	0.10 **				
Grade		0.71	0.83	0.08	[−0.93, 2.35]
Bullying Victimization		3.15	0.86	0.32 ***	[1.44, 4.86]
Step 2	0.07 *				
Gender		−2.38	1.13	−0.18 *	[−4.61, −0.15]
Witnessing Bullying		2.34	1.09	0.21 *	[0.25, 4.59]
Defending Behavior		0.31	0.65	0.04	[−0.98, 1.60]
Step 3	0.01				
Gender x Witnessing Bullying		−1.06	0.97	−0.13	[−2.97, 0.85]
Gender x Defending Behavior		−0.42	0.64	−0.06	[−1.68, 0.84]
Total *R*^2^	0.18 ***				

Note. *N* = 126. SE = standard error. CI = confidence interval. * *p* < 0.05, ** *p* < 0.01, *** *p* < 0.001.

**Table 4:** Hierarchical Regression Analyses for Social Distress and Avoidance.

Predictor	Δ*R*^2^	B	SE B	β	95% CI
Step 1	0.04				
Grade		−0.17	0.68	−0.02	[−1.51, 1.17]
Bullying Victimization		1.55	0.70	0.20 *	[0.75, 3.37]
Step 2	0.03				
Gender		−1.50	0.94	−0.14	[−3.36, 0.36]
Witnessing Bullying		1.11	0.91	0.13	[−0.70, 2.91]
Defending Behavior		0.16	0.54	0.03	[−0.92, 1.23]
Step 3	0.06 *				
Gender x Witnessing Bullying		−2.22	0.78	−0.33 **	[−3.77, −0.67]
Gender x Defending Behavior		0.53	0.52	0.09	[−0.50, 1.55]
Total *R*^2^	0.13 *				

Note. *N* = 126. SE = standard error. CI = confidence interval. * *p* < 0.05, ** *p* < 0.01.

**Table 5:** Hierarchical Regression Analyses for Fear of Negative Evaluation.

Predictor	Δ*R*^2^	B	SE B	β	95% CI
Step 1	0.08 **				
Grade		1.14	0.64	0.16	[−0.12, 2.40]
Bullying Victimization		2.06	0.66	0.28 **	[0.75, 3.37]
Step 2	0.05				
Gender		−1.94	0.87	−0.19 *	[−3.67, −0.23]
Witnessing Bullying		1.03	0.84	0.12	[−0.65, 2.70]
Defending Behavior		0.49	0.50	0.09	[−0.51, 1.48]
Step 3	0.04				
Gender x Witnessing Bullying		−1.14	0.74	−0.17	[2.60, 0.31]
Gender x Defending Behavior		0.98	0.49	0.18 *	[0.02, 1.94]
Total *R*^2^	0.17 **				

Note. *N* = 126. SE = standard error. CI = confidence interval. * *p* < 0.05, ** *p* < 0.01.

## Data Availability

Data are not available due to ethical restrictions.
